# Supplementary material for: Professional identity and its relationships with AI readiness and interprofessional collaboration
Source: PLoS One. 2025 May 16;20(5):e0322794. doi: 10.1371/journal.pone.0322794 (PMC12083793; doi:10.1371/journal.pone.0322794)
Supplement: Attachment — Submitted filename: Responses to Reviewer.docx [file pone.0322794.s002.docx]

Responses to Reviewer’s comments

Dear Editor:

We would like to extend our thanks for giving us a chance to resubmit the revised manuscript and to reviewers for reviewing the manuscript (PONE-D-24-39174), entitled “Professional Identity in the Age of AI and Interprofessional Collaboration among Healthcare Practitioners.” We reviewed the manuscript based on the editor and reviewer’s comments. We read and understood their comments, and we believe that they are fully addressed. All changes have been made and attached as a ‘Manuscript with Track Changes’.

| Reviewer’s Comment | Response | Reference |
| --- | --- | --- |
| After an initial evaluation, I commend your efforts to address the timely and relevant topic of professional identity in the evolving landscape of healthcare with the integration of artificial intelligence and interprofessional collaboration. | Thank you very much for your comments and efforts! |  |
| However, I have identified several areas that require significant improvement before the manuscript can be sent for peer review. Please find my detailed comments below: | Thank you for your thorough constructive feedback! |  |
| Flow and Readability: The manuscript seems to lack a coherent flow, making it challenging to follow the narrative. This could hinder reviewers' ability to assess the study effectively. Suggest restructuring the content to enhance clarity and logical progression. | The manuscript has been revised to address your comment. | Introduction and background. |
| Methodological Concerns: The reliability of the tools used, particularly the Medical Artificial Intelligence Readiness Scale (MAIRS) and the Readiness for Interprofessional Learning Scale (RIPLS), raises issues. For instance, some subscales have low Cronbach’s alpha values, which suggest poor internal consistency and may affect the credibility of the findings. Please justify the use of tools with low reliability scores or consider alternative validated instruments. | This issue was addressed by:  -Explanation for the use of the measure.  -reliability analyses of the study sample.  -and item-total correlation analysis was added.  The results demonstrated good to excellent reliability. | Methods-Measures and  Results sections |
| Data Availability: The data availability statement does not meet the PLOS ONE guidelines, as it should clearly indicate unrestricted access to data, barring exceptional cases. Ensure the data availability aligns with PLOS ONE’s policies, providing clear access to data or a valid justification for any restrictions. | Revised as  **Data availability statement:** The datasets generated and analyzed during the current study are available as a supplementary file accompanying this manuscript. |  |
| Specify Data Collection Period: The manuscript does not mention when the data was collected. This information is essential for understanding the study's context and potential relevance to the current state of the field. | Data collection occurred between November 14th, 2023, and February 13th, 2024. | Methods-Data Collection Procedure’s section. |
