# Supplementary material for: Professional identity and its relationships with AI readiness and interprofessional collaboration
Source: PLoS One. 2025 May 16;20(5):e0322794. doi: 10.1371/journal.pone.0322794 (PMC12083793; doi:10.1371/journal.pone.0322794)
Supplement: Attachment — Submitted filename: Responses to Reviewer 2.docx [file pone.0322794.s003.docx]

Responses to Reviewer’s Comments

Dear Editor,

We would like to extend our thanks for giving us a chance to resubmit the revised manuscript and to reviewers for reviewing the manuscript (PONE-D-24-39174), entitled “Professional Identity in the Age of AI and Interprofessional Collaboration among Healthcare Practitioners.” We reviewed the manuscript based on the editor and reviewer’s comments. We read and understood their comments, and we believe that they are fully addressed. All changes have been made and attached as a ‘Manuscript with Track Changes’.

Note: the title has been changed according to the reviewer’s comment to better reflect the nature of the study to:

“Professional Identity and its Relationships with AI Readiness and Interprofessional Collaboration”

| Reviewer’s Comment | Response | Reference |
| --- | --- | --- |
| Reviewer #1: |  |  |
| Dear Authors.  This is a very well written manuscript. | Thank you very much for your comments and efforts to improve the quality of the manuscript. |  |
| We recommend to compare your result to results by previous researchers. | Thank you. Highlighted in the discussion section. | Discussion |
| Reviewer #2: |  |  |
| Detail in spreadsheet.  1. The goal of this manuscript was to examine professional identity and its relationships with AI readiness domains and interprofessional collaboration components, however, the title “Professional Identity in the Age of AI and Interprofessional Collaboration among Healthcare Practitioners”, does not clearly address this point. It is recommended to revise the title. | Thank you for your comments and efforts to improve the quality of the manuscript.  We have revised the title to:  Professional Identity and its Relationships with AI Readiness and Interprofessional Collaboration | Title |
| 2. The manuscript does not mention literature evidence on the relationships between professional identity and AI readiness. This could be added in the background. | Thank you. Added to the background section. | Background  Paragraph 3. |
| 3. In RIPLS, items 10-12 are Negative Professional Identity and 13-16 are Positive Professional Identity，how to get “Professional identity scored a mean of 15.72 (SD = 3.75)” in line 245？Please provide more context or explain in detail. | Thank you very much for your valuable comment. The values were incorrect and have been replaced with the correct ones Mean = 11.93, SD = 2.93 | Results. |
| 4. line 210-216, Table 3. The preliminary analysis revealed that the professional identity scores were not normally distributed among the participants nor among the groups of participants which require nonparametric comparative analyses, why used Pearson r correlation coefficient to examine the relationships between professional identity and other variables (AI readiness and IPC components)? Why not the Spearman's rank correlation coefficient? | We appreciate the reviewer’s insightful comment regarding the choice of correlation analysis, which has allowed us to strengthen the methodological rigor of our study.  The analysis was redone using Spearman's rank correlation coefficient and reported in the results. | Results |
| 5. Line 166，”This questionnaire includes 17 items”, but according to reference 19, the Readiness for Interprofessional Learning Scale (RIPLS) includes 19 items. Please review carefully. | Thank you for highlighting this important point. The number is corrected. | Methods |
| 6. Line 167，”which are team teamwork and collaboration (T&C)”, it seems like the term “team” could be removed. | Thank you. Done. | Methods |
| 7. The "ai" in the line 252 should be capitalized. | Thank you. Done. | Results |
