# Supplementary material for: Professional identity and its relationships with AI readiness and interprofessional collaboration
Source: PLoS One. 2025 May 16;20(5):e0322794. doi: 10.1371/journal.pone.0322794 (PMC12083793; doi:10.1371/journal.pone.0322794)
Supplement: Attachment — Submitted filename: Responses to reviewers.docx [file pone.0322794.s005.docx]

Responses to Reviewer’s Comments

Dear Editor and Reviewers,

We would like to extend our thanks for giving us a chance to resubmit the revised manuscript and to reviewers for reviewing the manuscript (PONE-D-24-39174R3), entitled “Professional Identity and its Relationships with AI Readiness and Interprofessional Collaboration.” We reviewed the manuscript based on the reviewer’s attached Excel file. We read and understood their comments, and we believe that they are fully addressed. All changes have been made and attached as a ‘Manuscript with Track Changes’.

| Reviewer’s Comment | Response | Reference |
| --- | --- | --- |
| Abstract: Contain about 200 to 250 words. | Revised to adhere to the guidelines | Abstract |
| Abstract: Please add timeframe. We suggest to add quantitative values | Added to the abstract | Abstract |
| Discussions: We suggest to add some comparison with previous researches | Comparisons were added and highlighted | Discussion |

Thank you very much for your comments and efforts to improve the quality of the manuscript.

On behalf of the authors,

Wafaa Taan
